# Echo View Cells From Bio-Inspired Sonar

**DOI:** 10.3389/fnbot.2020.567991

**Published:** 2020-11-05

**Authors:** Jacob D. Isbell, Timothy K. Horiuchi

**Affiliations:** ^1^Department of Electrical and Computer Engineering, University of Maryland, College Park, MD, United States; ^2^Institute for Systems Research, University of Maryland, College Park, MD, United States; ^3^Program in Neuroscience and Cognitive Science, University of Maryland, College Park, MD, United States

**Keywords:** bat, echolocation, place cells, place fields, robotics, sonar, neural network, skim

## Abstract

Place recognition is naturally informed by the mosaic of sensations we remember from previously visiting a location and general knowledge of our location in the world. Neurons in the mammalian brain (specifically in the hippocampus formation) named “place cells” are thought to reflect this recognition of place and are involved in implementing a spatial map that can be used for path planning and memory recall. In this research, we use bat-inspired sonar to mimic how bats might sense objects in the environment and recognize the views associated with different places. These “echo view cells” may contribute (along with odometry) to the creation of place cell representations observed in bats. Although detailed sensory template matching is straightforward, it is quite unlikely that a flying animal or robot will return to the exact 3-D position and pose where the original memory was captured. Instead, we strive to recognize views over extended regions that are many body lengths in size, reducing the number of places to be remembered for a map. We have successfully demonstrated some of this spatial invariance by training feed-forward neural networks (traditional neural networks and spiking neural networks) to recognize 66 distinct places in a laboratory environment over a limited range of translations and rotations. We further show how the echo view cells respond between known views and how their outputs can be combined over time for continuity.

## Introduction

The hippocampal formation in the mammalian brain is well-known for its population of “place cells,” a type of neuron that responds when an animal is in a particular place in its environment. Studies in the rat suggest that these cells use internal odometry signals (allowing the system to operate in darkness) as well as external sensory cues (allowing the system to recognize places and correct the odometry system) (O'Keefe, 1976; Jung et al., 1994). In the flying, echolocating bat, neurons with very similar properties have been found (Ulanovsky and Moss, 2007; Yartsev et al., 2011; Yartsev and Ulanovsky, 2013; Geva-Sagiv et al., 2015). Unlike rats, bats have the uncommon ability to perceive the three-dimensional locations of objects by actively emitting sounds and localizing the reflections (Wohlgemuth et al., 2016), allowing the bat to navigate where other sensory systems, such as vision, are ineffective. Although the signal processing and neural mechanisms with which bats *recognize* places is still largely unknown, modeling this capability with biologically-plausible sensors and robotics can give us insights into problems that bats encounter and motivate future behavioral and neurophysiological experiments with bats.

Although most robotic explorations into mapping and navigation have focused on variants of the SLAM (simultaneous localization and mapping) algorithm using light-based sensors (e.g., computer vision or LIDAR) (Strösslin et al., 2005; Bachelder and Waxman, 2011) for metrically-accurate maps, little work has been done exploring how a bat might use sonar to accomplish the same task. One good example is that of Steckel and Peremans (2013) that used a biomimetic sonar device on a mobile, ground robot to navigate and map different office and laboratory environments, however, the SLAM algorithm used was not meant to be a model of a biological system. The work presented here addresses the question whether place cells can be recognized over an extended region using only a narrow-band (~40 kHz) sonar in a laboratory environment. Unlike the place cells that signal when the animal is in a particular area (i.e., the “place field”) based on a combination of odometry and sensory inputs, we are constructing “echo view cells” that recognize previously encountered views (i.e., an “echo fingerprint”) based solely on sonar. Phenomenologically similar to primate “spatial view cells” that are active when the animal is gazing at a particular set of objects (over a limited field-of-view), these echo view cells recognize previously memorized echo patterns. Unlike primate spatial view cells, however, object range is included in the pattern and thus the echo view cells fire over a small region of the environment.

A neural network model was used to implement echo view recognition that incorporates concepts from machine learning related to pattern separation and classification. A key aspect of this investigation is the attempt to bridge the gap from high-dimensional, low-level, sensory inputs to the more symbolic, discrete nature of place recognition that is critical to higher-level cognitive models of path planning (Koul and Horiuchi, 2019). A key goal is to ensure that the echo view cells respond over a wider area and not just to a single coordinate in space. One limitation of the work is that only limited information is available from the narrowband sonar (typical objects are represented by only a few echoes) and object recognition was difficult, preventing a landmark-based approach, as is common for visual place recognition algorithms. Instead, views were recognized based solely on the spatiotemporal pattern of echoes allowing the memorization of views in a variety of environments without prior training of an object recognition layer. From view recognition, direction-independent place recognition can be constructed in convergence with odometric information. Such approaches to place recognition with sonar have been used (Ollington and Vamplew, 2004; Vanderelst et al., 2016). One challenge with sonar is that small changes in the position and angle (particularly in man-made environments) can produce large changes in the resulting echo pattern. Multi-path reflections are also sensitive to positioning. To explore this, data was taken with a large variety of small changes to the positioning of the sonar.

This work explores two very different neural networks that can achieve this: a single layer neural network operating on a recorded echo pattern presented as an image, and a biologically-realistic, spiking neural network (SNN) presented with echoes in the time domain to simulate live sonar signals. In addition to our motivations to ultimately model and understand the biological implementation of sonar-guided behavior (mentioned above), this work has applications for mobile, autonomous robotics. There are many circumstances when a drone may need to navigate in a dark building for stealth, through a building filled with smoke, through a forest with dense fog, or through tunnels filled with dust. Since standard cameras and LIDAR do not work well in these environments, sonar is a reasonable alternative or complementary sensor. Sonar has been shown to be useful for obstacle avoidance (Eliakim et al., 2018). Currently, the most common use of sonar systems is underwater. Since the speed of sound is much faster underwater, the effective range and efficiency of sonar is greatly increased underwater. Current laser and radar systems consume much more energy than a sonar system; this would reduce the robot's field time and potential range (Jiang et al., 2010). The weight and cost of radar systems can also reduce their feasibility of use. One can imagine a lightweight, flying drone that can quickly maneuver through a dark house and provide a map based more on sensory features and not metrical details, closer to the way humans communicate with each other.

## Materials and Methods

### Hardware

**Figure 1:**
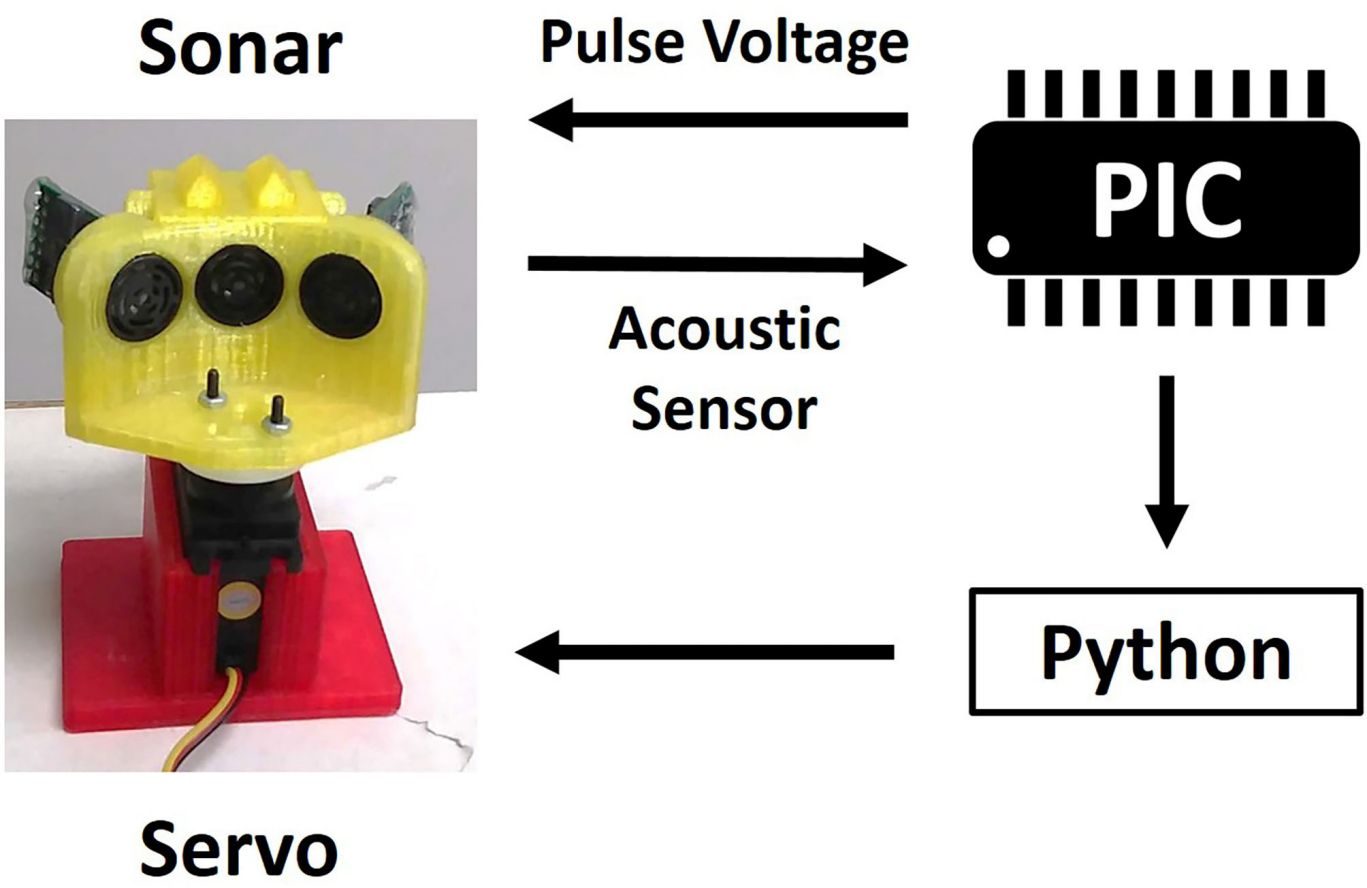
The flow of signals through the hardware. The microcontroller sends pulse voltages to the transducers and reads the acoustic voltage off the transducers. This data is sent to Python (van Rossum, 1995) on a PC, which also controls the servo motor.

**Figure 2:**
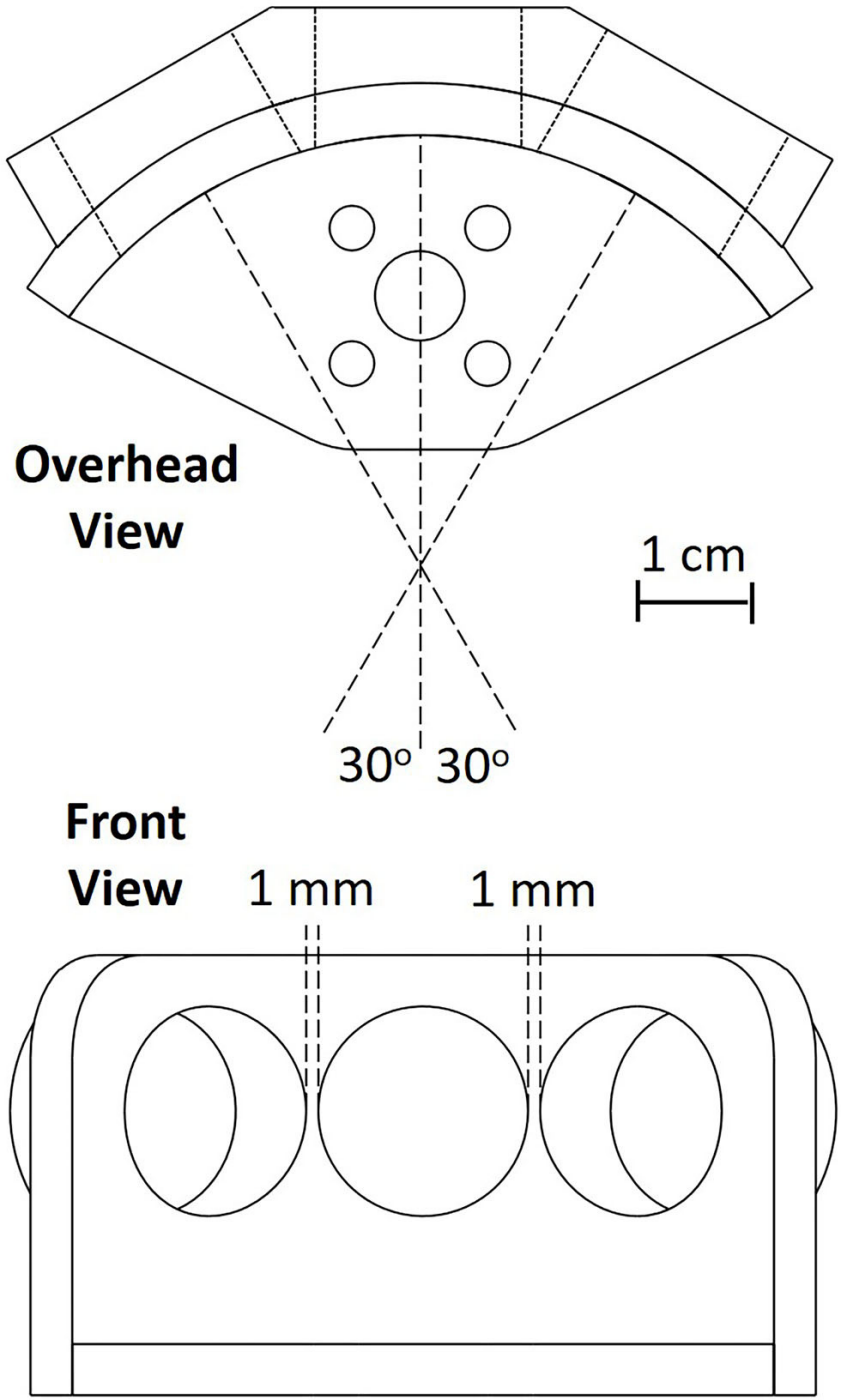
The schematic used for the 3-D printing of the sonar transducer holder. The sonar system consists of three sonar transducers oriented with 30 degree angular separation mounted on a rotating servo motor (not shown).

The sonar system used in the work presented here consists of three custom-modified MaxBotix® sonar transducers, similar to the MaxBotix XL-MaxSonar®-EZ™ commercial series of sonar range finders, a custom PIC® 18F2620 microcontroller-based sonar controller board, a Futaba S148 hobby servo, and a computer interface to both record and display echo signals and control the servo to orient the sonar (shown in Figure 1). The transducers act as both a speaker and a microphone. They resonate at 40 kHz and will only detect signals near this frequency. The custom sonar boards report a logarithmically-compressed envelope signal as an analog voltage. This compression allows the output to report the very wide dynamic range of amplitudes that occurs with sonar without saturating. The maximum working range of this sonar is 7.65 m. These transducers were custom modified to provide more control over the timing and duration of the outgoing pulse, a louder outgoing pulse, and access to a log-compressed envelope of the transducer response. All these functionalities are now commercially available through MaxBotix. The transducers are placed in a 3-D printed mount (shown in Figure 2) on the servo motor. In this demonstration system, the transducers transmit and receive over a cone of about +/– 30 degrees (−6 dB beam width), so the transducers are held facing 30 degrees apart to ensure sufficient overlap and coverage of the area in front of the transducers for binaural localization based on interaural level differences. The ultrasonic pulse trigger-timing and analog-to-digital (A/D) conversion is done by the microcontroller. The majority of the data processing is performed on the microcontroller to ensure a quick response.

**Figure 3:**
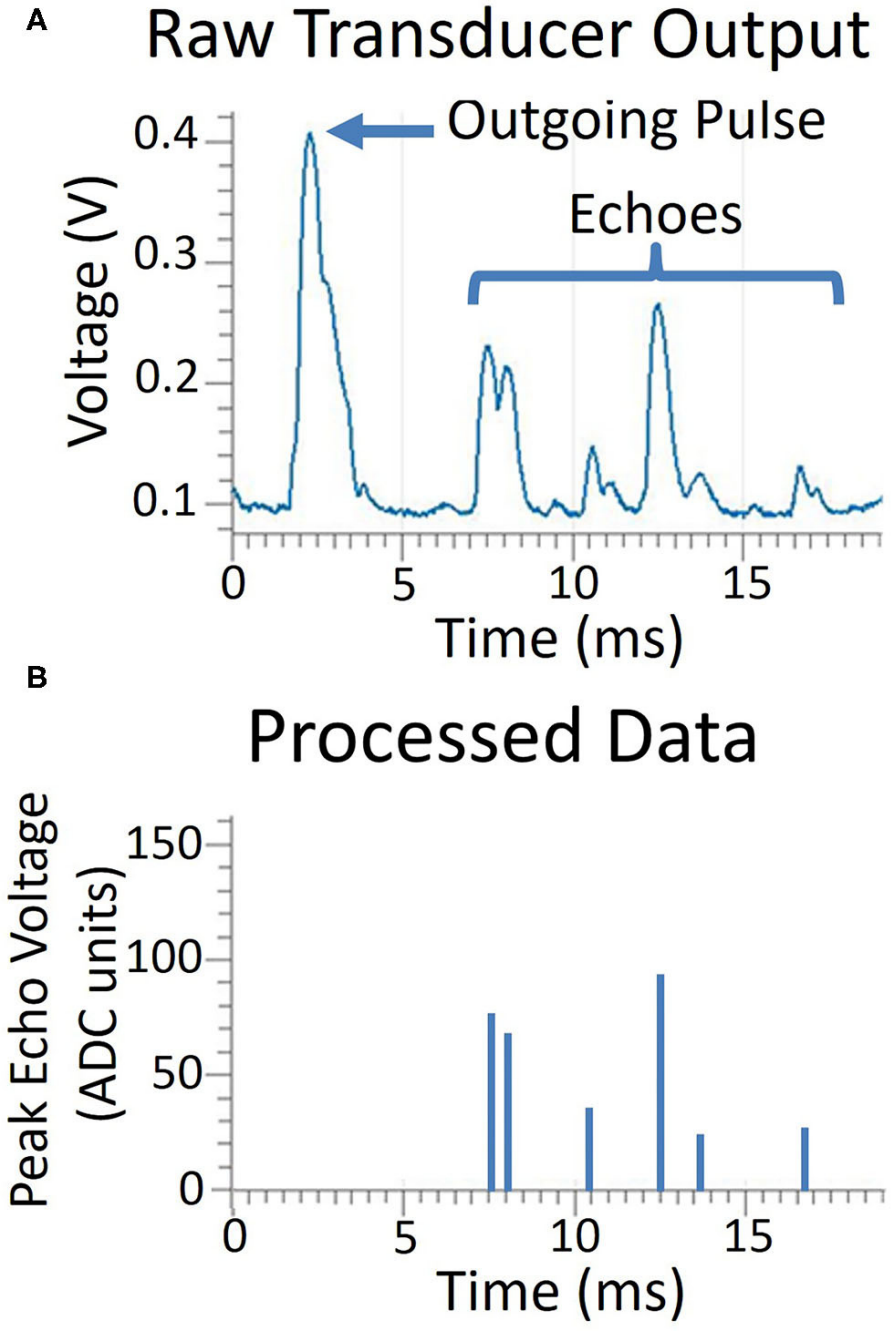
A comparison of the raw transducer data and the processing done on the microcontroller. In the top graph **(A)**, the transducer voltage is shown as it produces the outgoing pulse and receives the echoes. Only peak magnitude and time of echo peak are processed and recorded, shown in the bottom graph **(B)**.

The sonar system executes four steps: pulsing, sampling, processing, and communicating. A short duration pulse voltage (~0.25 ms) is supplied to the transducer, however, due to the resonant quality of the transducer, the emitted sound has a ring-up and ring-down period, resulting in an extended pulse duration of about 1 ms. Following the pulse, the transmitting transducer continues to ring for several milliseconds. Echoes can be detected during this ringing period once the amplitude has diminished sufficiently, so a short two millisecond delay is incorporated before sampling begins. The log-compressed envelope voltage is sampled every 8th of a millisecond, a sampling frequency of 8 kHz. An object is detected when the temporal derivative of the envelope switches from positive to negative, denoting a peak. The range is determined by finding the time when the envelope reaches its peak value. Envelope voltages on all transducers are recorded at the time of the peak. Our sampling time of an 8th of a millisecond gives us a range resolution of 2.14 cm or 0.84 in. We sample for 255 time bins, giving us a range of 5.5 m or 18 ft. Following the sampling period, echo data is transferred via serial interface to a PC and all further processing on the information is performed on the PC. An example of this data is shown in Figure 3.

The code used on our microcontroller is available at https://github.com/jacob-isbell/sonarPIC/blob/master/PICcode.asm.

### Dataset Description

**Figure 4:**
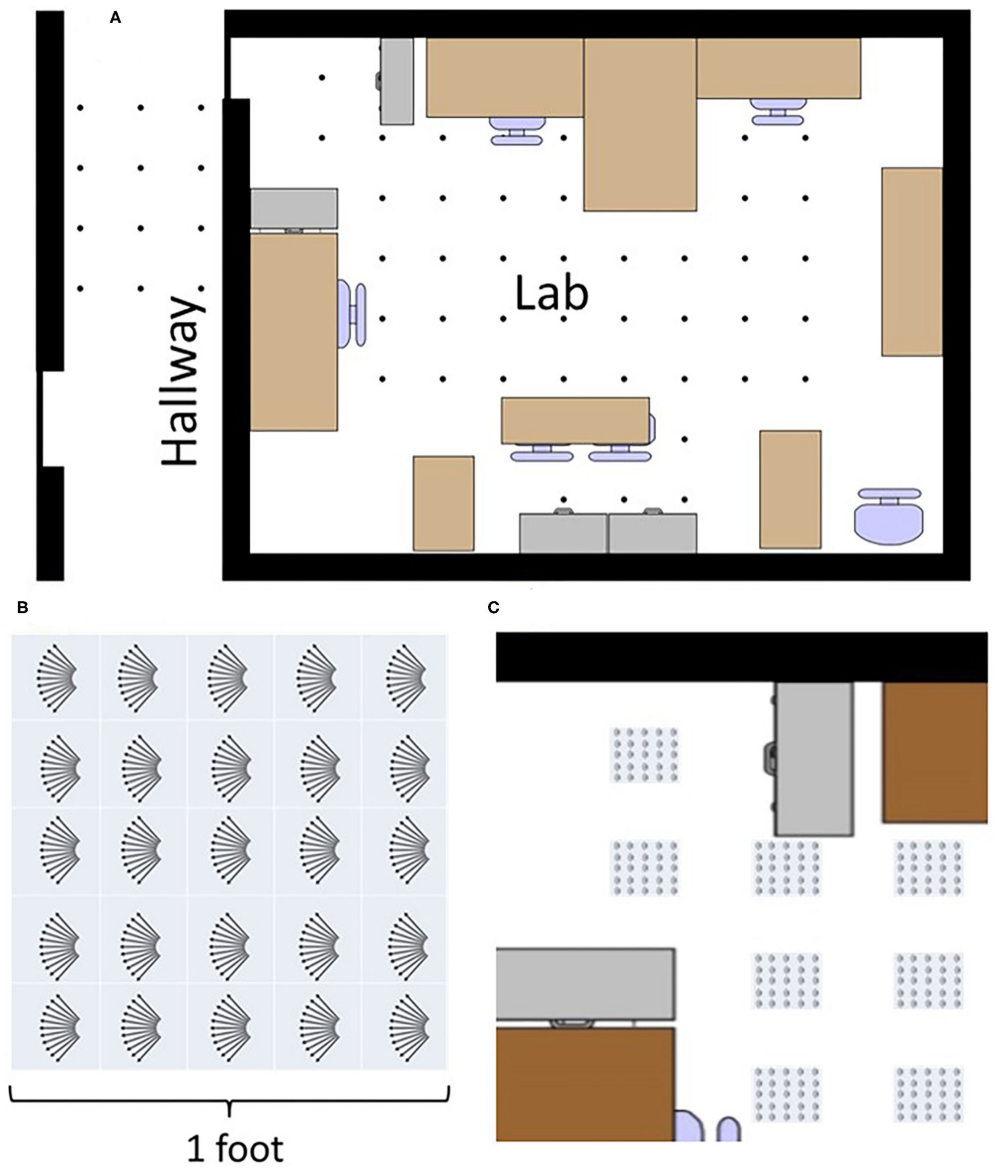
The top image **(A)** shows a map of places data was recorded. Every dot is a recorded place. Locations and objects are approximately to scale. Bottom left **(B)** shows how a variety of data was recorded at different translations and rotations at each point in the lab. Eleven angles were recorded along five rows and five columns giving 275 recordings at each place. Bottom right **(C)** shows explicitly how data was recorded at each place, capturing a large variety of data throughout the lab.

Data was recorded in our laboratory and the adjoining hallway. 66 different recording locations were spread throughout this environment. Locations were spaced 2 feet apart where possible, forming a grid-like placement (Figure 4A). No attempt was made to restructure the objects in the lab to accommodate the sensing; things were left as they were. No objects were moved during the recording at different locations.

To capture a broader view, a variety of data was collected at different translations and rotations within each square at each of the 66 locations. Across 1 square foot, data was recorded at 25 different translations inside a 5 × 5 square grid with a 3 inch (7.6 cm) spacing. At each of these 25 points, data was recorded at 11 different angles, ranging from −5 to +5 degrees in 1 degree increments (Figures 4B,C). Ten samples were taken at each angle. In total, each square location has: (25 translations) × (11 angles) × (10 repetitions) = 2,750 sonar images per location. With 66 locations, the full data set consists of 181,500 sonar images.

### Echo Fingerprint Recognition

Two different neural network architectures were tested for their ability to recognize which of the 66 locations a sonar pulse came from. A conventional, single layer network was used and a biologically-plausible, temporally-based architecture called the Synaptic Kernel Inverse Method (SKIM) (Tapson et al., 2013) was used. The inputs and outputs of both networks were similar. The inputs consisted of one sonar image. 255 range bins were used with data from the 3 transducers, resulting in a 765-dimensional input vector. The envelope amplitude data was supplied to the network. If there was no echo in a time bin, the value was kept as zero. The resolution of each range bin was 2.14 cm or 0.84 in. Each sonar image was L2 normalized before being fed to the network. While normalizing means the network doesn't have direct access to the echo magnitudes, the relative magnitude between echoes contains more reliable and reproducible information, such as the magnitude difference between transducers which relates to echo direction. Each output corresponds to a different location, so with 66 locations there are 66 outputs. In both networks, a form of supervised learning was used to train the network.

Although the angle of an arriving echo could be calculated using the magnitude difference between the transducers (e.g., using interaural level differences) to reduce the dimensionality, we chose to retain the raw values and let the network learning rules determine how this information would be used.

#### Single Layer Feedforward Network

**Figure 5:**
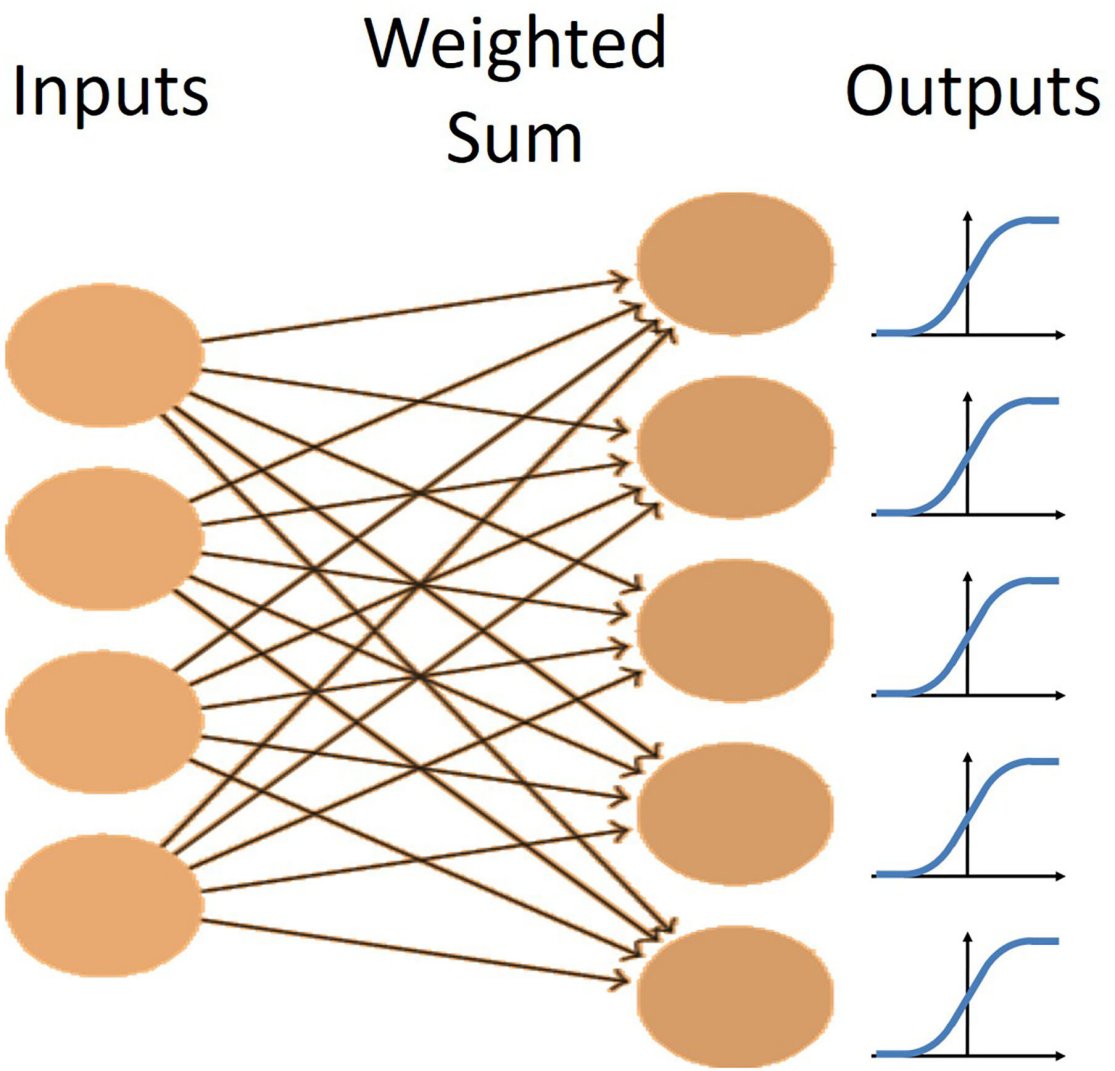
The network architecture for the single layer network. There is one layer of fully connected weights from the inputs to the outputs. Each output has a logistic non-linearity applied to it to maintain outputs between 0 and 1.

In this experiment, a very simple neural network was used to process the data. The network consisted of the input layer fully connected by weights to the output layer (Figure 5). The non-linear logistic function was applied to the summation of weighted inputs to provide the output. Learning was performed by a modified version of gradient descent that uses an adaptive momentum term to speed learning, called the AdamOptimizer algorithm (Kingma and Ba, 2014). This was implemented in the machine-learning software package, TensorFlow (Abadi et al., 2015) on Google's Colaboratory cloud computing platform (Bisong, 2019), allowing us to speed up the training with free use of their GPUs.

In this task, the single layer network performed as effectively as multiple-layer networks and its simplicity led to an easier observation and analysis of how the network was solving this problem.

#### Synaptic Kernel Inverse Method (SKIM)

**Figure 6:**
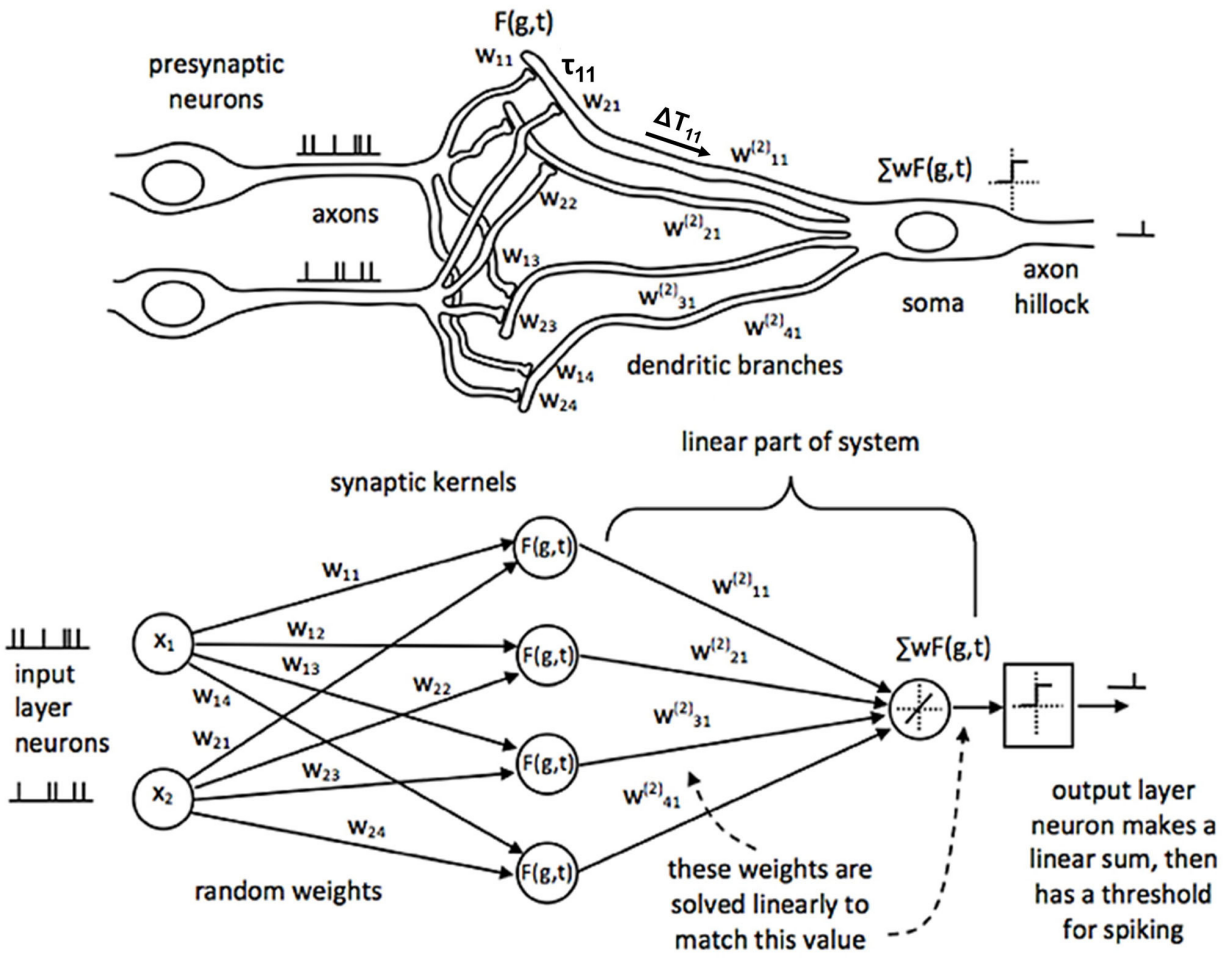
Adapted from Tapson et al. (2013). The architecture for the SKIM neural network. The top of the figure shows what a corresponding biological system would look like, while the bottom shows this network from a computational perspective. Inputs from the presynaptic neurons are summed onto the dendrites of the postsynaptic neurons. Each dendrite has an associated non-linear, synaptic kernel [*F*(*g,t*)] with a time constant (τ), and dendritic delay (ΔT). The dendritic activity is summed onto the soma and creates a spiking output when above a threshold. The weights from the input layer to the hidden layer are static (w_xy_); the linear connection from the hidden layer to the output has weights that are trained (${\text{w}}_{\text{yz}}^{\left(2\right)}$).

SKIM is a multi-layer network architecture that combines the benefits of Extreme Learning Machines (ELM) but with spiking neuron (temporal) representations. Sonar lends itself to being represented in the spiking domain because echoes themselves are inherently time-based signals and typically pulsatile in nature. The temporal nature of this network suggests a real-time implementation using spiking neuromorphic hardware (Moradi et al., 2017). Figure 6 illustrates the SKIM network architecture (Tapson et al., 2013).

**Figure 7:**
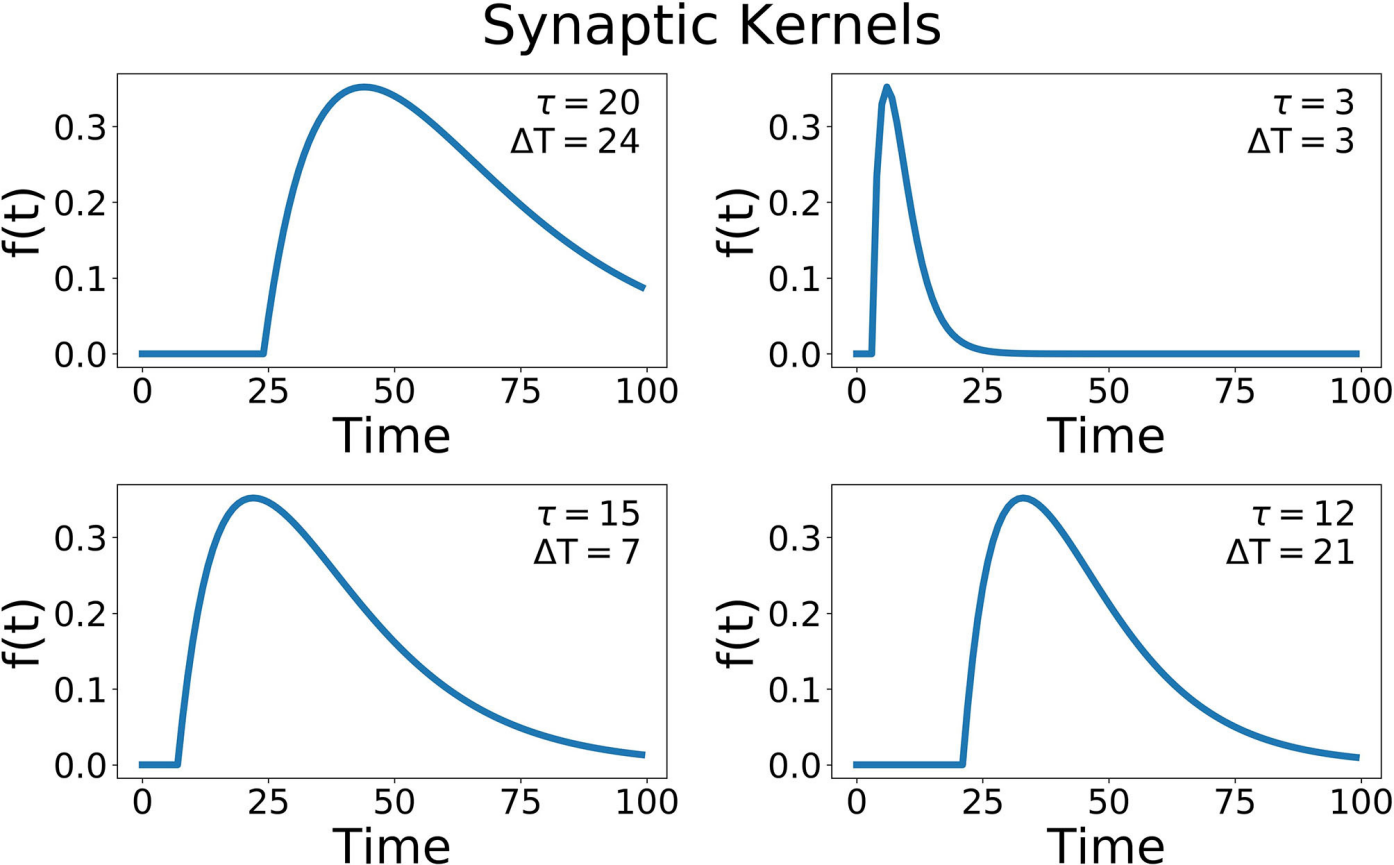
Some example synaptic kernels. Two parameters are changed, the delay for the onset of the function (ΔT), and the width of the alpha function (τ). The x-axis corresponds to the variable t of this function.

The first layer of weights in the SKIM network consists of fixed, random weights connecting the inputs to the hidden layer. These weights can be positive or negative. The fanout here is usually 10–20 (or a hidden layer that has 10–20 times more neurons than the input layer), resulting in a very large hidden layer. This is typical of an ELM approach, which aims to expand the dimensionality of the input data to make pattern separation easier (Huang et al., 2011). There is also a non-linearity applied at each hidden unit. Every hidden unit has a randomly selected temporal synaptic kernel associated with it that consists of a time delayed alpha function. If A is the activation of the unit, t is the time, Δ*T* is the delay, and τ the width of the alpha function, the equation is:

$$\begin{array}{l} f(t)=tanh(A\;\frac{t-\Delta T}{\tau }e^{-\frac{t-\Delta T}{\tau }}) \end{array}$$

where different hidden units have different delays (Δ*T*) and widths of the alpha function (τ) (Figure 7). The time delay is essential to recognizing patterns that occur over time, and gives the network a form of memory, a way to be influenced by data in the past. A compressive non-linearity (the hyperbolic tangent, tanh) is applied as well. These hidden units create a high-dimensional, non-linear transformation of the input data that has occurred recently in time. This allows for complex, temporal patterns to be more easily recognized and separated.

The next layer of this network is linear. There are a set of fully-connected weights from the hidden layer units to the output. These are the weights that are modified during learning. Since this is the only dynamic part of the network, the learning is simplified. As this is a linear transformation with a known hidden-unit activation and a known output (since we are performing supervised learning), the weights can be solved for analytically.

If *M* is the number of hidden units and *k* is the number of time steps in our dataset, we obtain a matrix describing the hidden unit activation over time, *H* ε R ^M*xk*^. If *N* is the number of outputs, we have the output activation matrix, *Y* ε R ^N*xk*^. The weights connecting the two layers will be *W* ε R ^N*xM*^, such that *WA* = *Y*. To find the weights we simply have to solve for *W*, giving *W* = *YA*^+^, where *A*^+^ can be found by taking the Moore-Penrose pseudoinverse of A.

To solve this analytically, we use the Online PseudoInverse Update Method (OPIUM) (Tapson and van Schaik, 2013). This is an application of Greville's method, which shows an incremental solution to finding the pseudoinverse, but is adapted and simplified for this specific problem to reduce the needed computation without losing accuracy.

## Results

### Single Layer Network

**Figure 8:**
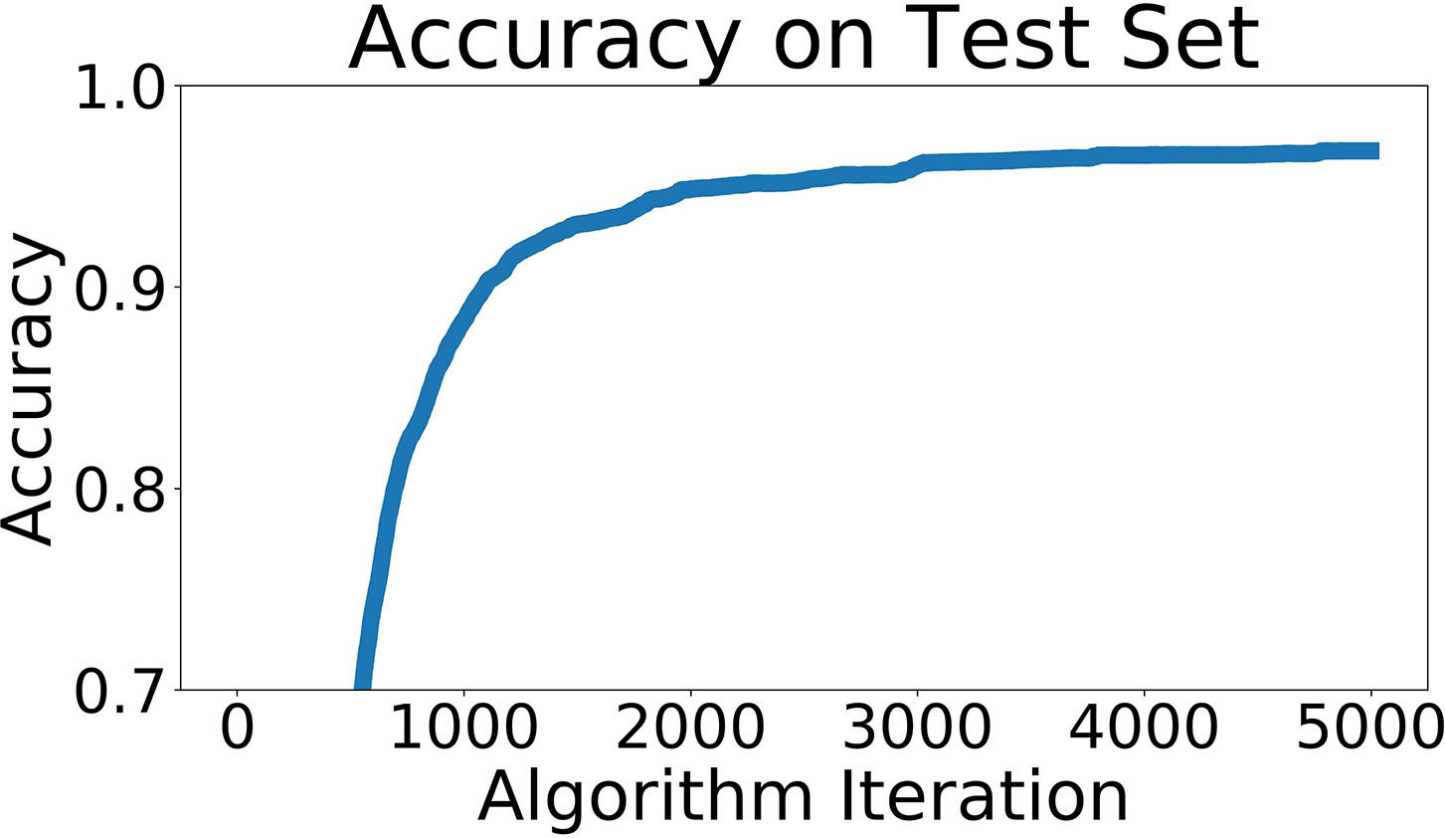
Network accuracy as training progresses. Each algorithm iteration takes ~0.5 s, and the network takes about 1 h to train.

This network was trained to predict which of the 66 locations a sonar pattern came from. The recorded sonar dataset was split into three parts, 80% training data, 10% testing data, and 10% validation data. The data was randomly shuffled across locations and positions within locations before being split into these three groups. Our accuracy of identifying the location of a particular pattern from the validation data set reached 97.5%. A graph of the accuracy across the training regimen is shown in Figure 8.

**Figure 9:**
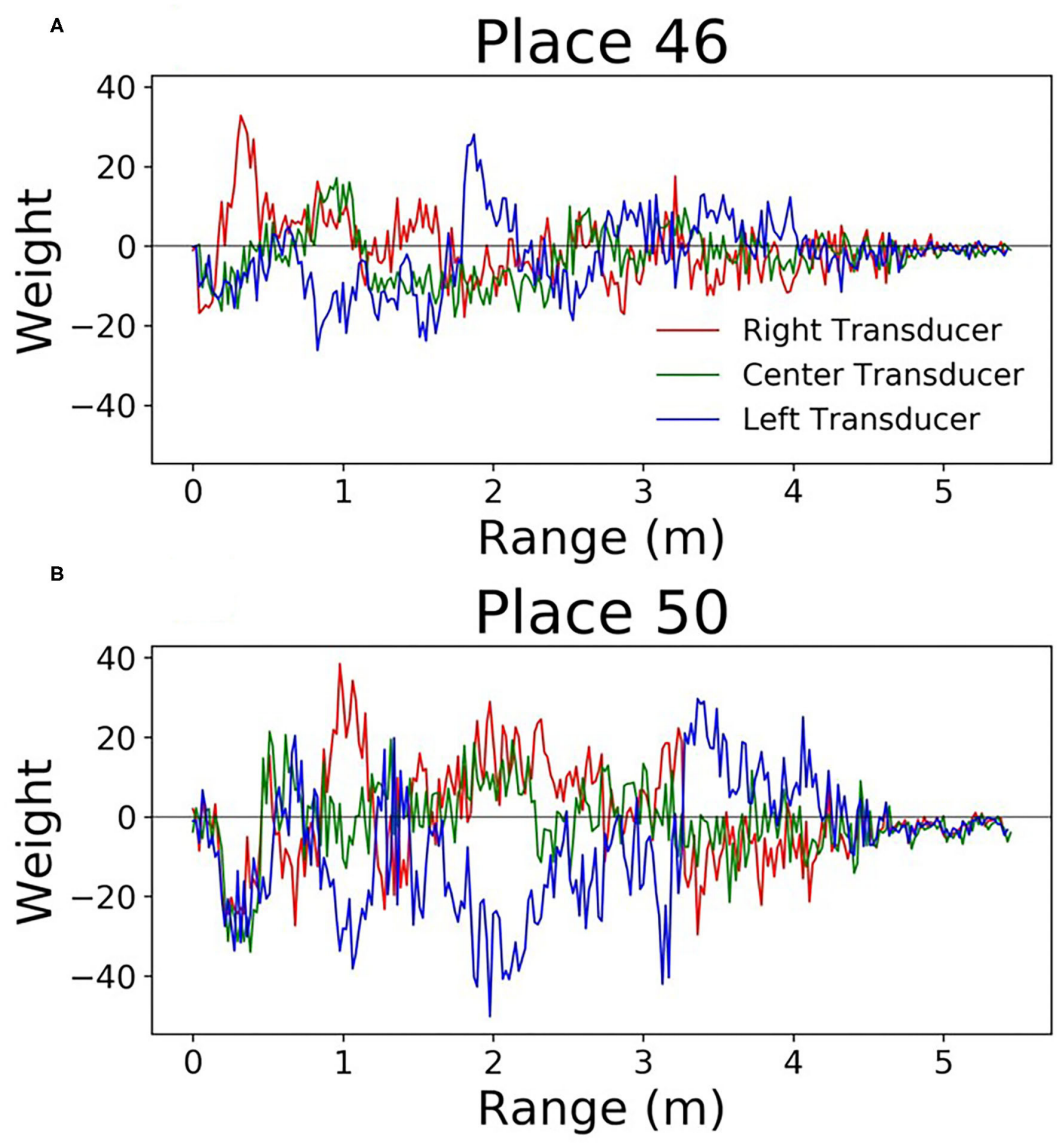
Perceptive fields of the output neurons in the single layer network **(A,B)**. It's clear that in some spots these perceptive fields split the left and right signals. This gives the network the ability to discriminate direction.

**Figure 10:**
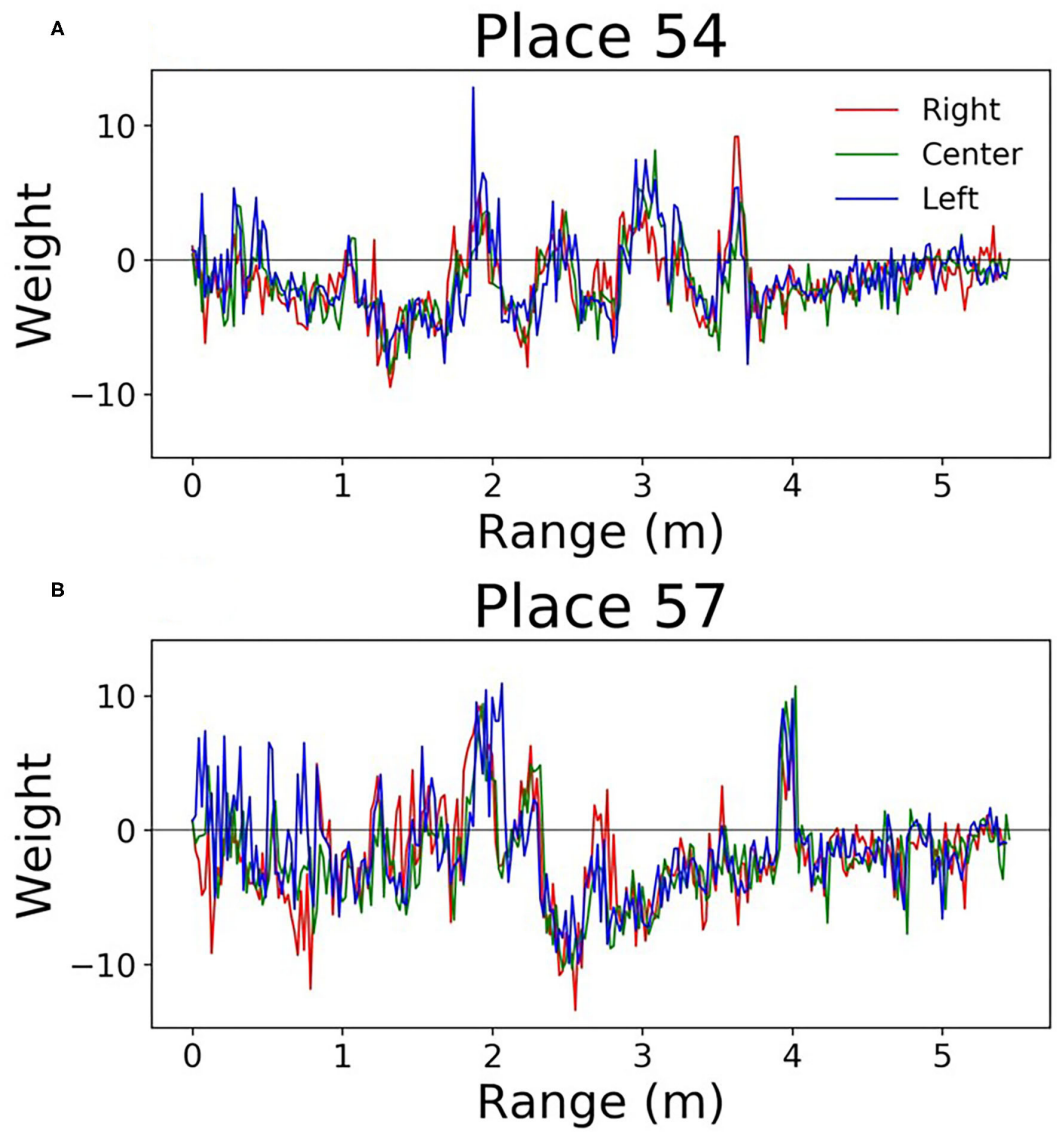
Perceptive fields of the output neurons in the single layer network. These **(A,B)** are from the hallway data. These weights were of lower amplitude, and all transducers were correlated with one another.

Since this network is very simple, it is easy to understand how the weights can be interpreted. Each output neuron has a weight corresponding to every input. These can be thought of as the perceptive field of this output neuron. By looking at which inputs cause the output to activate, we can get an idea of the sonar image preferred by each output neuron. Figure 9 shows some example weights from the network. One noticeable pattern in these weights is the splitting that occurs between the right and left transducers; there are clear ranges where one will be positive and the other will be negative. Functionally, this is the network learning to look for objects at a certain angular orientation. Another clear pattern that arose in the network weights; the weights from the hallway seemed to be synchronized across transducers (Figure 10). These weights were also lower in amplitude than those from inside the lab.

**Figure 11:**
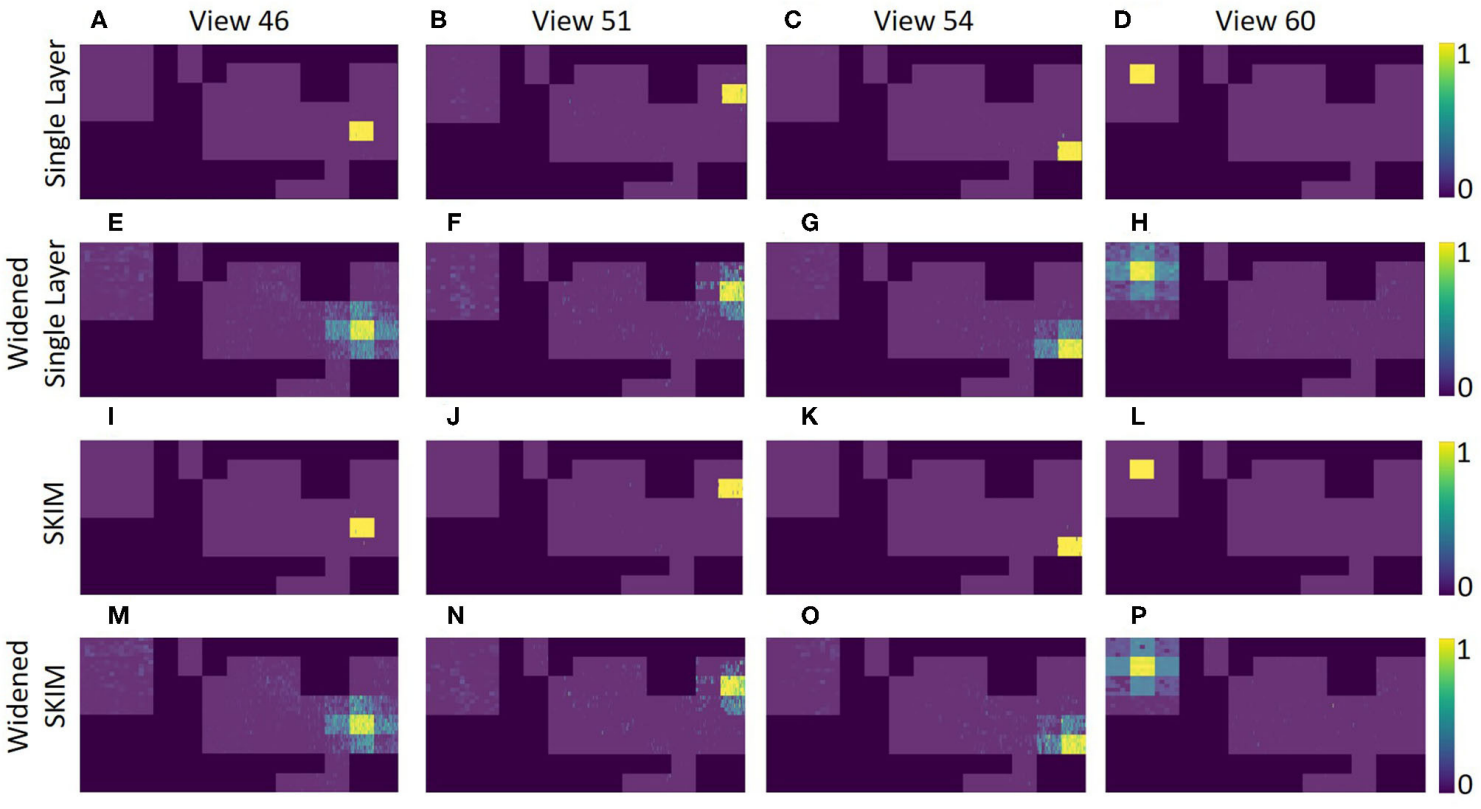
An overhead view of the different echo view fields created by the two networks. This map is the same as shown in the top of Figure 4. Each plot represents a different echo view cell's activation across the entire map. The top plots **(A–D)** show the original network for four different views, and the plots **(E–H)** shows the single layer network with widened labels, resulting in neighboring views being activated. Plots **(I–P)** show the view activations for the SKIM network and the widened SKIM network. It is important to note that only areas in the training dataset are displayed. The 1 foot squares in between each of the locations have been omitted (shown explicitly in Figure 4C).

Figures 11A–D shows how the different view cells responded across the whole map. It is clear that the network learned very rigid boundaries where it was trained to do so. Although this demonstrates a successfully trained network, the sharp distinctions between neighboring locations is not what is seen in mammalian place cells.

### SKIM

In the SKIM network trained with OPIUM, we achieved up to 93.5% accuracy on our dataset. The choice of time constants (τ, the alpha function widths) and delays (ΔT) for the synaptic kernels was very important. The time constants determine the temporal precision the network can observe; large time constants lead to less temporal precision. Long time constants provide tolerance to temporal jitter between patterns but result in a loss of temporal discrimination when needed. The time constants used for this network covered one to five time bins, with τ′s randomly chosen between 0.5 and 1.5, keeping a relatively narrow and precise response. The choice of delays determined which temporal part of the data is relevant (i.e., beginning, middle, end of the pulse). The delays were distributed randomly over the length of the sonar pulse to ensure that all the echoes had an equal probability of activating the network, with ΔT′s randomly chosen between 0 and 255. The network was trained to deliver an output at the end of a sonar image (*t* = 255). Accuracy was determined by taking the output neuron with the highest activation at t = 255. Figures 11I–L shows how the SKIM view cells responded across the whole map. The response is very similar to the single layer network with rigid boundaries between views.

### Recognition Outside of Training Data

**Figure 12:**
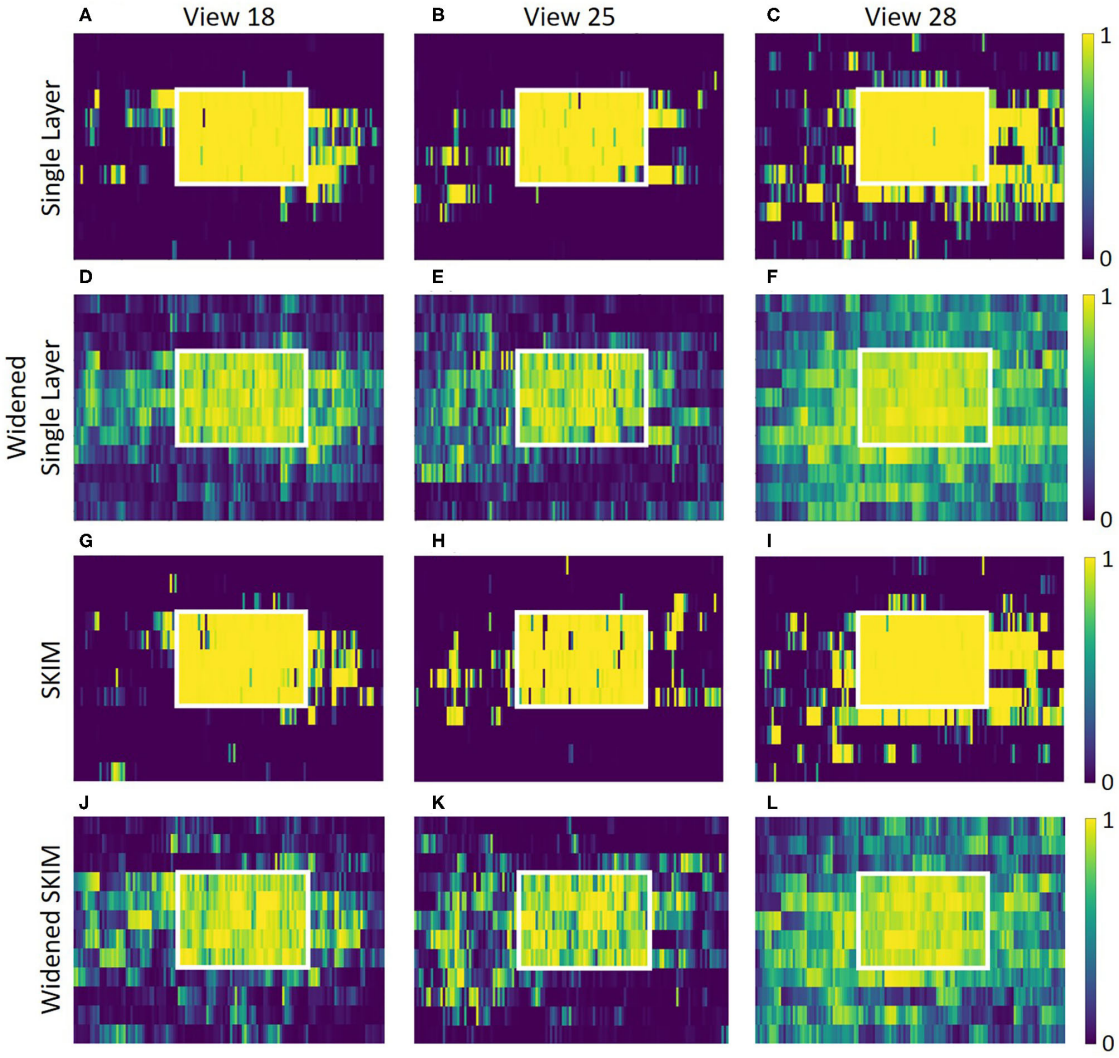
Each graph presents an overhead view of a location. Inside the white square is where training data was recorded; outside the white square is an adjacent area that was not used for the training of the networks. Along the x axis, eleven adjacent pixels show the eleven angles for each of the 25 (5 × 5) spatial positions inside the white box. Pixels along the y axis are spaced evenly. These view neuron activation patterns are generated by the corresponding output neuron from the neural network. The top plots **(A–C)** show how the single layer network responds around these locations, showing sparse activation outside the trained square and very high activation inside the square. Plots **(D–F)** show the single layer network trained to respond to neighboring views. Plots **(G–L)** show the same information, but for the SKIM network and the widened SKIM network. The widened networks show a much more spread out activation in the non-trained area outside the square.

Outside of the locations (squares) where data was collected, both networks does not predictably recognize that it is near a known location. The accuracy was high when in an area it was trained on, but recognition drops quickly even inches away. Figures 12A–C shows this for the single layer network; Figures 12G–I shows this for the SKIM network. To spread the activation of the network to neighboring areas outside the training area, network training was changed. Instead of an output neuron being trained to 1.0 in its corresponding location and all other neurons trained to 0.0, neighboring neurons were trained to respond to neighboring views. A Gaussian function was used, giving adjacent views an activation of 0.5 and diagonal views and activation of 0.38. After this round of training the accuracy of the single layer network dropped to 92.3%, while the accuracy of the SKIM network remained stable at 93.4%. Figures 12D–F,J–L show the results of this new training for the single layer network and SKIM network, respectively. The new activation pattern of the network is now spread through areas that were not explicitly trained on, and qualitatively looked more like biological place fields. Figures 11E–H,M–P also shows how these new view cells respond across the whole map. There is now more noticeable activation in areas that were not trained on. The cells have become much more broadly tuned. We call this new network the “widened” network, in contrast to the “original” network. The single layer network and the SKIM network responded very similarly in all the cases presented.

## Discussion

### Functionality Test Along a Path

**Figure 13:**
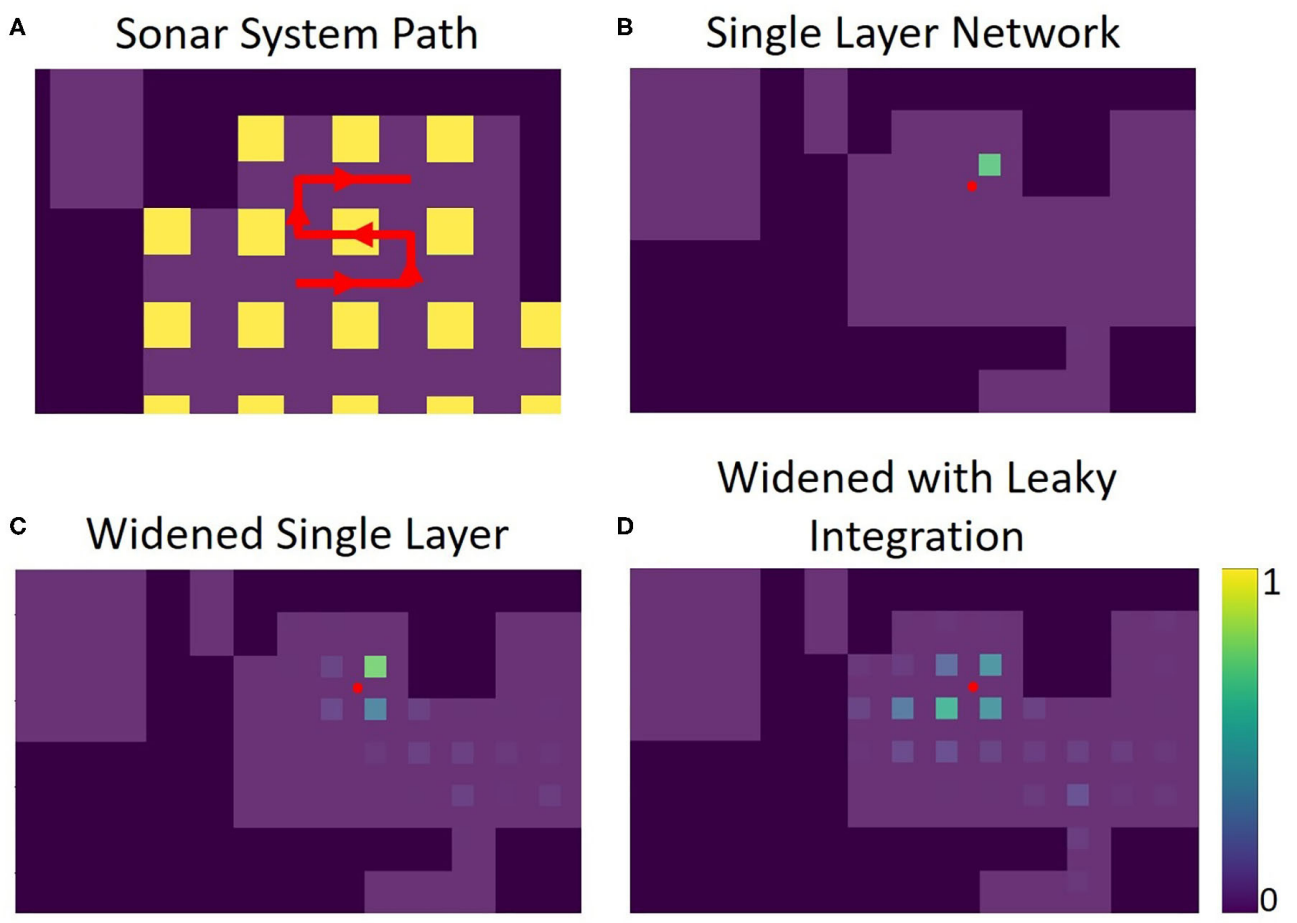
Panel **(A)** shows the path the sonar system moves through, in red. There are 39 positions total along this path, each position 3 inches from the last. The portion of the path within the yellow squares is contained in the training set for the networks (5 of the path positions). The rest of the path was not used for the training of the networks. Panels **(B–D)** show echo view field responses on the path. The red dot represents the position of the sonar. The activations of the echo view cells are shown in their corresponding location, seen as colored squares on the plots. The single layer network was trained to have only one view cell active at a time. The widened network allows for more cells to be active at once, improving accuracy in between trained views. The leaky integration maintains a more stable activation due to its use of the past activations in the path.

To demonstrate how this system might be used in practice, sonar data was recorded along a path consisting of points both inside and outside of the training data. The single layer network's response to this data shows how views can be recognized along the entirety of this path (Figure 13).

The widened network, which allows multiple view neurons to be active at once, creates a broader, more spatially-continuous response when compared with the original network. Less reliance on a single view neuron activating provides a more stable and nuanced interpretation of location. In situations where the original network fails to activate the correct view neuron, the widened network is more likely to alleviate the situation by activation of other nearby view neurons.

Leaky integration was also used to help smooth out the network response over time; each activation is given an exponentially decreasing tail over time. With A_t_ as the activation for a position at time t, and L_t_ as the activation for a position after leaky integration is applied, the equation used is **L**_**t**_**=αA**_**t**_**+**(**1−α**)**L**_**t−1**_. In this example, one view is about 10 movements wide. Using a leaky integration constant (**α)** of 5/9 allows for activation to be maintained at %10 of its original value 10 time steps in the future, allowing persistent activation while moving across a position at the cost of a slight lag. An equivalent way to calculate this would be to have each activation exponentially decaying over time; the corresponding time constant would be 4.5. In some locations on the path, the sonar is not able to correctly recognize the view. For this example, integration over time gives the network more stability and accuracy. Supplementary videos show the activations of the original network, the widened network, and the leaky integration applied to the widened network similar to Figure 13, but over the entire path.

The widened network with leaky integration gives consistently accurate results over the whole path. The echo view fields activated are generally smooth over space and decaying activation can be seen multiple locations away. To evaluate the effectiveness of these echo view fields, we calculated the activity-weighted centroid at each point on the path, giving us an average point of each field to compare with the actual position of the sonar. The distance between the activity-weighted centroid and the actual position was used to calculate a mean error. Across 117 steps along three different paths, the original network's average error was 28.6 inches (72.6 cm), the widened network's average error was 18.6 inches (47.2 cm), and the widened network with leaky integration's average error was 16.3 inches (41.4 cm). This system successfully recognized locations that are not contained in the training set; the network can generalize and recognize many nearby views. When this fails, leaky integration allows past information to maintain a stable sense of place for the system.

### Context/Previous Studies

These results complement previous studies that have used sonar to aid in place recognition. A large inspiration for our project was BatSLAM (Steckel and Peremans, 2013), a biomimetic sonar system that used odometry and sonar to map an area of their laboratory. Because odometry is quite inaccurate due to wheel slippage and other errors, such as compounding inaccuracies in estimating direction and position, sonar was used to provide error correction. Their system first drew paths of motion based solely on odometry. When the sonar-based recognition system recognized the current location from a prior visit, it updated the odometry system to match its memory and propagated the correction to earlier time steps for consistency. This was sufficient to correctly create a map of the area with little error. While this approach showed that sonar was able to aid place recognition, it did not do so in a biologically-plausible manner. Over the robot's path, 6,000 sonar measurements were taken, and 3,300 different places were established. While this system provides a method to maintain an estimate of the robot's position, it does not seem to reflect what little is known about how biological memories of the environment. Memorizing 3,300 different places all within one environment is computationally and memory-intensive; it is not a biologically-plausible algorithm. While our study attempted to show that odometry is not needed for view recognition, incorporating odometric information can provide a strong framework for unsupervised mapping. For example, a new “place” can be created when a system, using odometry, estimates it is a certain distance from any other “place.”

Another recent paper explored the idea of recognizing place with sonar in three different locations (Vanderelst et al., 2016). Using a very precise sonar sensor they measured the echo response at positions over a wide range of angles and along a linear, 10 m long path. They collected an enormous amount of data (over 20,000 echo traces) and evaluated whether the echoes varied smoothly over angle and distance as well as whether unique locations could be classified. Most of the data came from angular variation; large translational steps contrast the high angular resolution. They also found places that were difficult to distinguish between, mainly in open areas with few objects to sense, but concluded that sonar is enough to recognize most locations. When they were comparing different positions along a linear path, they compared the same precise angle (0.1 degree error) from the different positions. This is much more precise than an animal can hope to achieve, in reality both angle and position will be changing at the same time. We have shown in this study how sensitive an echo signature can be to changes in angle; we expect place recognition to be tolerant to moderate changes in the sensing direction. Our study can complement this one by providing a wider, two-dimensional range of positions for comparison as well as removing the need for very precise angular measurements.

In our study, all views were looking in the same direction. A network that could respond to views in different directions but at the same general location would be a step toward modeling a more general place cell. This could be modeled using an additional layer of a neural network. We have shown that different views can be separately recognized in a single layer network, another layer would be able to select which views correspond to the same place. This could be as simple as an “or” function that allows a view from any direction to activate the place cell.

### Single Frequency vs. Broadband

One important aspect of the sonar currently used in our system that is not biologically-realistic is the use of a single frequency (40 kHz). Bats use a broadband sonar pulse that provides much richer echo signatures with spectral content that likely contributes to object characterization that is not possible with our sonar (Mogdans and Schnitzler, 1990). Even with this limitation, this study shows that place field generation is still possible knowing only object range (inferred by the peak sound pressure on the three transducer channels) and echo magnitude. Different objects with multiple close surfaces can also produce echoes with different durations. With a broadband sonar sensor, it may be possible to significantly improve the size and reliability of the place fields.

## Conclusion

We have presented a robotic sonar system that uses ultrasonic transducers to mimic bat echolocation and have demonstrated two different networks that can recognize sonar views over a range of angles and offsets (“echo view fields”), with one network showing that this can be done in a biologically plausible manner. This view-based approach that does not require the identification of specific objects or explicit use of landmarks. The echo view cells produce “reasonable” responses outside of places where training data was collected and has the potential to be integrated into a larger system to model bat hippocampal place cells and spatial mapping.

## Data Availability Statement

The raw data supporting the conclusions of this article will be made available by the authors, without undue reservation.

## Author Contributions

TH: created the hardware and software template for the sonar system. JI: created the sonar mount and modified the software for this project, and performed the data collection and analysis with advice and guidance from TH. Both authors discussed the results and contributed to the final manuscript.

## Conflict of Interest

The authors declare that the research was conducted in the absence of any commercial or financial relationships that could be construed as a potential conflict of interest.

## Supplementary Material

Supplementary Video Similar to Figure 13, these videos show echo view fields responses along the path. The red dot represents the position of the sonar. The activations of the echo view cells are shown in their corresponding location, seen as colored squares on the plots. Video 1 shows the single layer network, which was trained to have only one view cell active at a time. Video 2 shows the widened single layer network, which allows for more cells to be active at once, improving accuracy in between trained views. Video 3 shows the leaky integration applied to the widened network, which maintains a more stable activation due to its use of the past activations in the path.
